# Specialist palliative care is associated with reduced healthcare utilization in patients with advanced esophageal and gastric cancer: a nationwide register-based study

**DOI:** 10.1007/s00520-025-09587-3

**Published:** 2025-06-05

**Authors:** Pauliina Kitti, Anu Anttonen, Mikko Nuutinen, Timo Carpén, Tiina Saarto

**Affiliations:** 1 Department of Radiotherapy, Comprehensive Cancer Centre and University of Helsinki, Helsinki, Finland; 2 Nordic Healthcare Group, Helsinki, Finland; 3 Department of Palliative Care, Comprehensive Cancer Centre and University of Helsinki, Helsinki, Finland

**Keywords:** Esophageal cancer, Gastric cancer, Palliative care, Healthcare, Home care

## Abstract

**Background and purpose:**

Esophageal and gastric cancer patients, with poor prognoses and complex symptom burdens, require comprehensive end-of-life care. This study evaluated the impact of specialist palliative care (SPC) on end-of-life healthcare utilization.

**Material and methods:**

We retrospectively analyzed nationwide healthcare utilization data for all adults who died of esophageal or gastric cancer in Finland in 2019, using National Health and Social Care Registers. Patients were compared based on timing of first SPC contact: early (> 30 days before death) or no/late (≤ 30 days).

**Results:**

The cohort included 732 patients (median age 72 years), with 233 (32%) having SPC contact, including 156 (21%) with early SPC. Most patients (79%) had late/no SPC. The median time for first SPC contact was 120 days before death for early SPC group, and 12 days for no/late group, *p* < 0.001.

Early SPC group, compared to no/late SPC, had fewer emergency department contacts (44% vs. 60%, *p* < 0.001) and secondary care hospitalizations (32% vs. 61%, *p* < 0.001) in the last month. Early SPC increased access to hospital-at-home (56% vs. 6%, *p* < 0.001) and SPC wards (19% vs. 4%, *p* < 0.001). Patients with early SPC died more often in SPC wards (19% vs. 4%,*p* < 0.001). Overall, 122 (17%) received hospital-at-home care, and were more likely to die at home (19% vs. 11%,*p* = 0.011) or in SPC wards (15% vs. 5%, *p* < 0.001).

**Conclusions:**

Few patients with advanced esophageal or gastric cancer receive SPC. Early SPC was associated with reduced healthcare utilization and improved access to SPC services, highlighting the importance of timely SPC initiation.

**Supplementary Information:**

The online version contains supplementary material available at 10.1007/s00520-025-09587-3.

## Introduction

Palliative care aims to reduce symptom burden [1] and improve quality of life at the end of life [2], but despite the international recommendations to integrate palliative care into cancer care early during the disease trajectory [1, 3], the duration of palliative care has been much shorter in practice. According to a recent meta-analysis the median time from the initiation of palliative care to death in cancer patients was just 15 days [4].

In patients with various advanced cancers, early palliative care contact has been shown to improve quality of life, reduce the risk of aggressive end-of-life care, hospitalizations, emergency department (ED) visits and hospital deaths [5–8]. The information is scarce on implementation of palliative care in patients with esophageal and gastric cancer. In a recent study, palliative contact was established in only 16% of hospitalized patients with esophageal cancer [9]. In our previous single center study of patients with esophageal and gastric carcinoma, the decision to terminate anticancer treatments i.e. palliative care decision, was either made during the last month of life or not made at all for half of the patients, leading to higher use of secondary hospital services and increased number of deaths at secondary hospitals [10].

The aim of this study was to evaluate frequency and timing of specialist palliative care (SPC) in esophageal and gastric carcinoma patients, as well as the impact of SPC on the utilization of healthcare resources at the end of life and the place of death.

## Material and methods

### Study cohort and data collection

The study cohort was retrospectively collected from the Causes of Death Register (Statistics Finland) and consisted of adults with esophageal or gastric cancer (International Classification of Disease (ICD-10) codes C15-C16) who died in Finland in 2019. Patients under 18 years and patients who died outside of Finland were excluded. The data originally comprised 733 patients, but one who died outside Finland was excluded, resulting in a final cohort of 732 patients. The date and place of death were also documented.

Data on healthcare and social service utilization were extracted from The National Care Register and Kanta Services. These mandatory registries are maintained by the Finnish healthcare authority. The registers provide data on socio-demographics, healthcare and social service utilization, advance care directives, and opioid prescriptions. The data covered all clinic visits, contacts, and care periods within primary, secondary, and tertiary healthcare as well as social service facilities, including long-term care institutions. For this study, secondary and tertiary healthcare data were combined under the category of secondary healthcare. Emergency care included contacts to both primary and secondary emergency departments. Specialist palliative care utilization was tracked using specific healthcare unit codes. The ICD-10 code Z51.5 indicates a palliative care decision, referring to situations in which disease-stabilizing or -modifying treatments are limited to improve prognoses, and the focus is on comfort and palliative care. The first occurrence of the Z51.5 code in the Care Register data was considered the date of the initial statement of the palliative care decision. The data collection was carried out from the beginning of 2018 until the end of 2019. Personal identification numbers were used to link the data, which were later pseudonymized with research identifiers. The study was conducted and reported in accordance with the STROBE (Strengthening the Reporting of Observational Studies in Epidemiology) guidelines.

### Specialist palliative care contact

The SPC service includes outpatient clinics, consultation teams, hospital-at-home service and SPC wards and hospices. Place of death was reported as SPC if the patient was under SPC on the day of death. The utilization of healthcare resources in the last month was compared between two patient groups based on the timing of their first SPC contact: 1) early contact (31 days or more) and 2) no contact or late contact (30 days or less before death). These groups were chosen based on previous studies suggesting that a timeline of less than one month is considered insufficient to provide the benefits of palliative care [11, 12].

### Ethical statement

The study was conducted in collaboration with the Finnish Institute for Health and Welfare (THL) as part of the Project on Quality Information on Palliative Care and End-of-life Care. The study was approved by THL Dnr: 12,345,556. In accordance with Finnish legislation for research, no additional ethics committee approval was required.

### Statistical analysis

Descriptive statistics, including medians, means, ranges, frequencies, and percentages were utilized. Group comparisons were conducted using Fisher’s exact test and Chi-square, or the Mann–Whitney test when distributions were not equal. A logistic regression model was used to compare age, gender and SPC contact with the use of ED or hospitalizations in the last month. Statistical significance was set at p < 0.05. IBM SPSS Statistics version 29 (SPSS Inc., Chicago, IL, USA) was used for data analysis. The statistical analyses were done by two of the authors (P. K. and M.N).

## Results

**Fig. 1:**
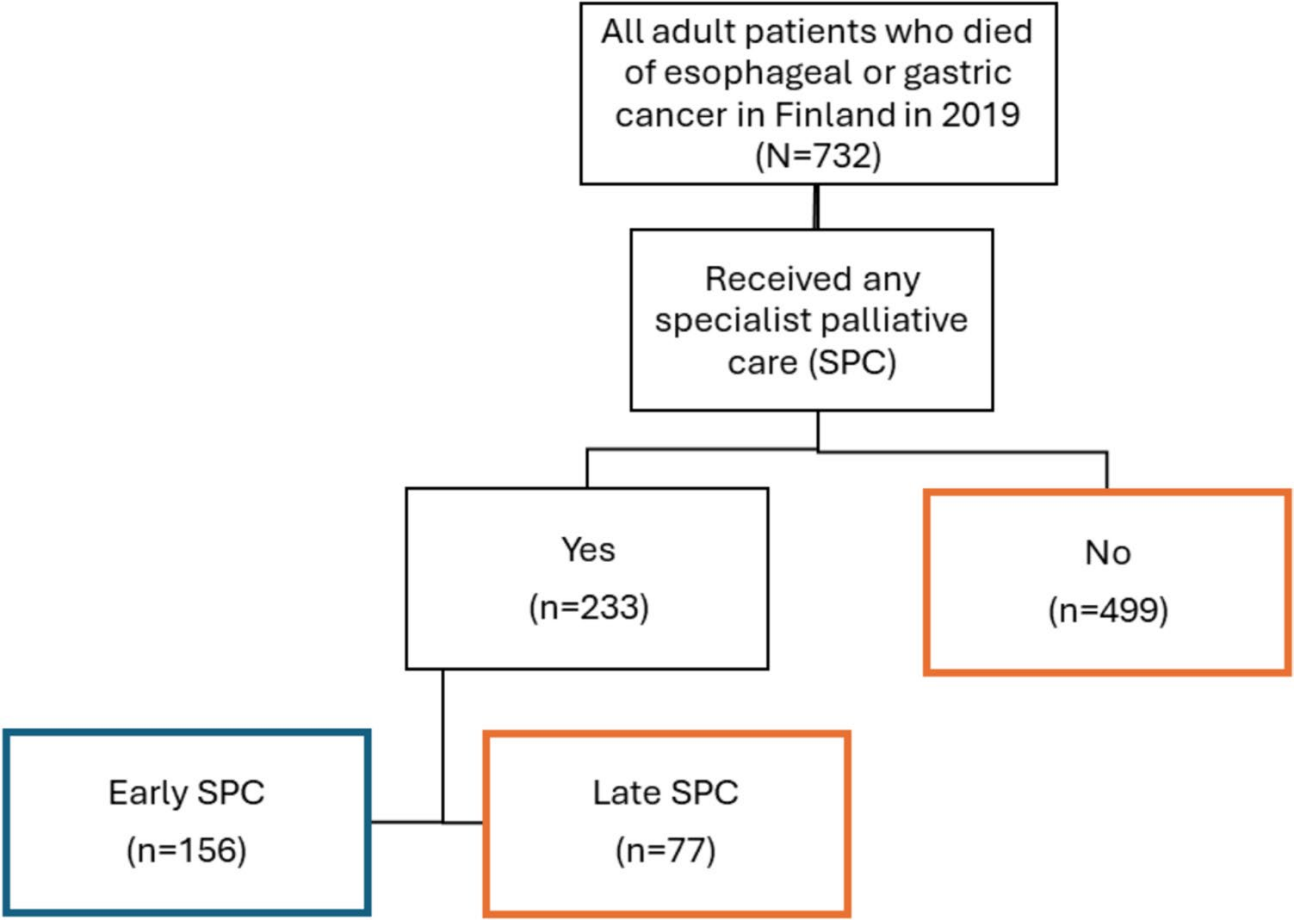
Patient flow chart

**Fig. 2:**
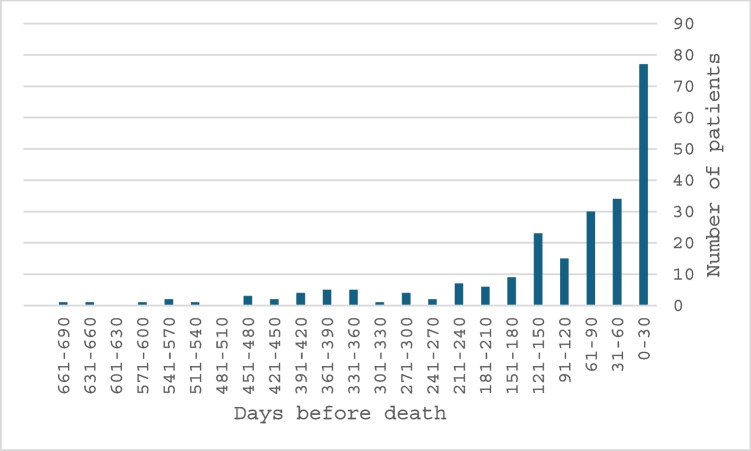
Distribution of timing of the first specialist palliative care contact

The cohort consisted of 732 patients, of whom 317 (43%) had esophageal cancer and 415 (57%) gastric cancer. Patient characteristics are shown in Fig. 1 and Table 1. Patients were categorized into two groups based on the timing of first SPC contact: (1) Early SPC and (2) No/Late SPC. This grouping was guided by preliminary results (see Supplement, Fig. 1), which showed similar healthcare utilization patterns between the No and Late SPC groups, as well as between the Early and Very Early (> 90 days before death) SPC groups. The contact to SPC was established for 233 patients (32%). Of these, for 156 patients (21% of the whole cohort) SPC contact was established early (> 30 days before death). Median time for the SPC contact was 64 days before death, but the largest peak occurred within the last 30 days of life. The timeline of the first SPC contact to death is displayed in Fig. 2. Patients with early SPC were slightly older (75 years vs 72 years, *p* = 0.009) compared to the no/late group.

**Table 1:** Patient characteristics

	All *n* = 732	Early SPC *n* = 156	No/late SPC *n* = 576	*p*
Female (%)	264 (36%)	57 (37%)	207 (36%)	0.480
Median age (range)	72 (31–102)	75 (31–96)	72 (33–102)	0.009
Type of cancer				
Esophageal cancer	317 (43%)	72 (46%)	245 (43%)	0.236
Gastric cancer	415 (57%)	84 (54%)	331 (58%)	0.236
SPC contact				
No	499 (68%)		499 (87%)	< 0.001
Yes	233 (32%)	156 (100%)	77 (13%)	< 0.001
Late (30 days or less)	77 (11%)	0	77 (13%)	
Early (> 30 days)	156 (21%)	156 (100%)		
31–90 days	64 (9%)	64 (41%)		
91 days or more	92 (13%)	92 (59%)		
The median time for the SPC contact before death in days (IQR)	64 (22–140)	120 (64–211)	12 (7–22)	< 0.001
ICD-10 diagnosis code Z51.5 Palliative care	408 (56%)	118 (76%)	290 (50%)	< 0.001

*IQR* Interquartile range, *SPC* specialist palliative care, Early SPC: more than 30 days before death, No/late SPC: 30 days or less before death

**Fig. 3:**
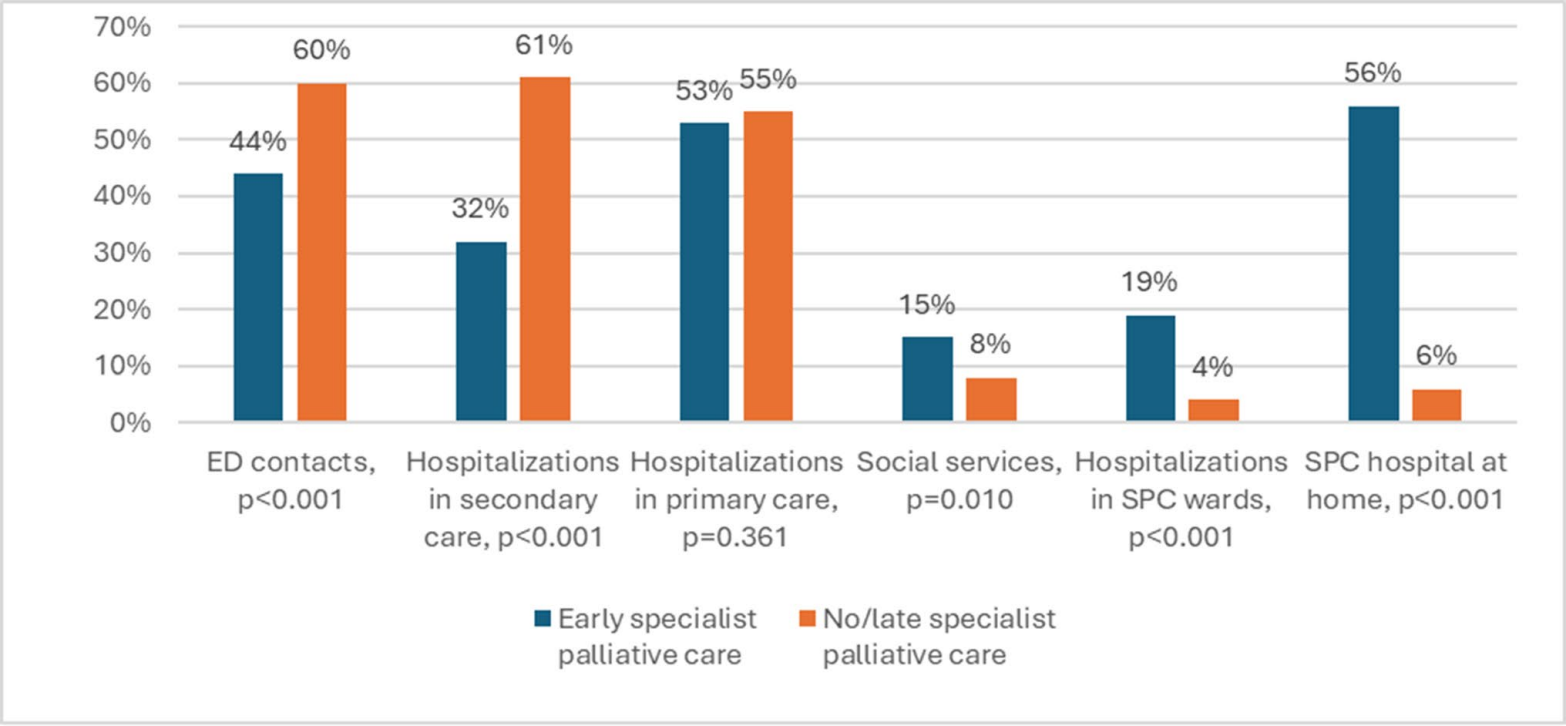
Impact of specialist palliative care (SPC) on healthcare resource utilization. ED: emergency department

In the last month of life, 56% of all patients had ED contacts, 55% were hospitalized in secondary care and 55% in primary care hospitals. Patients with early SPC had fewer ED contacts (44% vs 60%, *p* < 0.001), secondary healthcare hospitalizations (32% vs 61%, *p* < 0.001) and secondary outpatient contacts (38% vs 67%, *p* < 0.001) compared to patients with no or late SPC, respectively. No difference was found in utilization of primary healthcare services during the last month of life (hospitalizations 53% vs 55%, *p* = 0.361; outpatient contacts 64% vs 65%, *p* = 0.370) (Table 2 and Fig. 3).

**Table 2:** Comparisons of healthcare utilization in the last month of life based on timing of first specialist palliative care (SPC) contact

	All *n* = 732	Early SPC *n* = 156	No/late SPC *n* = 576	*p*
Emergency department contacts	412 (56%)	68 (44%)	344 (60%)	< 0.001
Outpatient contacts				
Secondary healthcare	443 (61%)	59 (38%)	384 (67%)	< 0.001
Primary healthcare	475 (65%)	99 (64%)	376 (65%)	0.370
Hospitalizations				
Secondary healthcare	400 (55%)	50 (32%)	350 (61%)	< 0.001
median number of inpatient days (range)	6 (1–30)	4 (1–26)	7 (1–30)	0.042
Primary healthcare	401 (55%)	83 (53%)	318 (55%)	0.361
median number of inpatient days (range)	12 (1–30)	15 (1–30)	12 (1–30)	0.157
SPC	233 (32%)	156 (100%)	77 (13%)	< 0.001
Hospital at home	122 (17%)	88 (56%)	34 (6%)	< 0.001
Specialist palliative care outpatient unit	73 (10%)	40 (26%)	33 (6%)	< 0.001
SPC ward	53 (7%)	30 (19%)	23 (4%)	< 0.001
Home care	284 (39%)	76 (49%)	208 (36%)	0.003
Social services	69 (9%)	23 (15%)	46 (8%)	0.010
Place of death				
Home	88 (12%)	22 (14%)	66 (12%)	0.221
Hospital-at-home support	21 (3%)	15 (10%)	6 (1%)	< 0.001
Hospital	608 (83%)	127 (81%)	481 (84%)	0.305
Hospital, other than SPC ward	557 (76%)	98 (63%)	459 (78%)	< 0.001
SPC ward	51 (7%)	29 (19%)	22 (4%)	< 0.001
Long term care	36 (5%)	7 (5%)	29 (5%)	0.486

*SPC* specialist palliative care, Early SPC: more than 30 days before death, No/late SPC: 30 days or less before death

In Table 3, the regression analyses show that SPC contact was significantly associated with both hospitalizations and ED contacts. Patients with no or late SPC contact had a higher likelihood of hospitalizations (OR 3.822, 95% CI 2.556–5.715, *p* < 0.001) and ED contacts (OR 1.847, 95% CI 1.284–2.657, *p* < 0.001) compared to those with early SPC contact. Age at death was significantly associated with a reduced likelihood of both hospitalizations (OR 0.955, 95% CI 0.939–0.972, *p* < 0.001) and ED contacts (OR 0.976, 95% CI 0.963–0.989, *p* < 0.001), indicating that older patients were less likely to require these services in the final month of life. Men had a lower likelihood of hospitalizations (OR 0.666, 95% CI 0.454–0.977, *p* = 0.038) and ED contacts (OR 0.708, 95% CI 0.518–0.968, *p* = 0.030) compared to women.

**Table 3:** Different background factors explaining Hospitalizations (A) and the Emergency Department contacts (B) during the last month of life in logistic regression analysis

A) Hospitalizations				
	*n*	OR	(95% CI)	*p*
Age at death	732	0.955	0.939–0.972	< 0.001
Gender				
Female	264	ref		
Male	468	0.666	0.454–0.977	0.038
SPC contact				
Early	156	ref		
No/Late	576	3.822	2.556–5.715	< 0.001
B) Emergency department contacts				
	*n*	OR	(95% CI)	*p*
Age at death	732	0.976	0.963–0.989	< 0.001
Gender				
Female	264	ref		
Male	468	0.708	0.518–0.968	0.030
SPC contact				
Early	156	ref		
No/Late	576	1.847	1.284–2.657	< 0.001

In the last month of life, 39 patients (25%) with early SPC visited the oncology outpatient unit and 10 (6%) were hospitalized in oncology ward as compared to 259 (45%) and 92 patients (16%) with no/late SPC (*p* < 0.001 and < 0.0001, respectively). The number of hospitalizations and ED contacts were highest during the last week before death. especially in patients with no or late SPC contact (Supplement, Fig. 2).

Majority of the patients (76%) died in a hospital. Early SPC contact was associated to a lower likelihood of hospital death (63% vs. 80%, *p* < 0.001, respectively) (Table 2).

Of all the patients, 122 (17%) received hospital-at-home service. Patients with hospital at home were more likely to die at home (23 patients [19%] vs. 65 [11%], *p* = 0.011) or in SPC wards (18 patients [15%] vs. 33 patients, [5%]; *p* < 0.001). They were less likely to be hospitalized in secondary care (42 patients, [45%] vs. 358 patients, [56%]; *p* = 0.025) or die in a hospital (74 patients, [61%] vs. 483 patients, [79%]; *p* < 0.001) compared to those without hospital-at-home service.

## Discussion

SPC was rarely present in the treatment of patients with esophageal or gastric carcinoma despite the advanced stage of the disease. The early onset of SPC, median 4 months before death, reduced the utilization of secondary healthcare and the rate of hospital deaths. However, the rate of hospital deaths remained high in our nationwide cohort of deceased patients with advanced esophageal or gastric carcinoma.

In our cohort, every third (32%) of the patients received SPC, but still one-third of these patients were referred to a palliative care unit within the last month before death, a timeframe potentially too short to achieve meaningful benefits. In this study, early palliative care was defined as initiation no later than 30 days before death. In the literature, the definition of “early palliative care” varies, ranging from 30 days [13], to 3 months [14, 15], and up to 3 to 6 months [16]. In previous studies, the proportion of patients with early palliative care contact (defined as 3 months before death) have ranged from 10 to 33% [16, 17]. Our findings are consistent with these data, as 13% of the patients had SPC contact at least 3 months before death. While the association of SPC with utilization of healthcare services was evident when SPC contact occurred at least one month before the death, the median time for the first SPC contact in early SPC group was 120 days prior to death, supporting earlier initiation of SPC.

During the last week of life, hospitalizations in secondary healthcare and ED contacts increased remarkably in our study, which can be explained by progressive deterioration of the patient as the terminal phase of life approaches. This exponential increase in hospitalizations has been also previously reported; for instance, one fifth of Belgian patients are hospitalized in the final week [18]. In previous studies, 43–71% of cancer patients were hospitalized during their last month [19–21]. Similarly, 34% of cancer patients have ED visits in the last two weeks [22], and 26–61% in their last month [21].

In our study, early SPC contact was associated with reduced utilization of secondary healthcare services, including inpatient hospitalizations, outpatient visits and ED contacts in the last month of life. The lack of difference between early or no/late palliative care groups in primary healthcare hospitalizations, was at least partly due to the fact that primary healthcare hospitals were mainly responsible of end-of-life care due to lack of dedicated SPC wards in Finland. In regression analysis, the association with early SPC and reduced secondary healthcare hospitalizations and ED contacts was confirmed. In regression analysis, older age was also associated with reduced hospitalizations, possibly reflecting more aggressive cancer care in younger patients at the end of life.

In randomized clinical trials focusing on effect of early integrated palliative care, no differences in healthcare resource utilization have been found [8, 23], although primary outcomes have been quality of life. Temel’s landmark study [6] demonstrated a reduction in aggressive end-of-life care (defined as no or late hospice care or chemotherapy within last two weeks of life) for patients with early integrated palliative care. While the study did not have adequate power to examine specific indicators of aggressive care, there appeared to be fewer hospitalizations and ED visits in the last 30 days. In previous retrospective studies the early initiation of palliative care has reduced aggressive end-of-life care across various cancer types [16, 17, 24]. Measures of overly aggressive cancer care include hospital deaths and events in the last 30 days, such as ED visits, hospitalizations, intensive care unit visits, chemotherapy administration [25–27]. In our study chemotherapy use or ICU was not measured, but in other measurements, cancer care was less aggressive in patients with early SPC. These findings support the early initiation of SPC for patients with esophageal and gastric cancers.

Overall, SPC reduced ED contacts and secondary care hospitalizations; however, in the terminal phase, home-based services such as hospital at home are often necessary. Patients with early SPC contact were more often enrolled in hospital at home, home care and social services, which support care at home. Interestingly, hospital-at-home service was associated with increased likelihood to dying at home or in a SPC ward and decreased likelihood of hospitalizations and death in a hospital. Our findings are in line with the previous Cochrane reviews [28, 29] reporting that hospital at home increased the number of patients who died at home, and also seemed to be less expensive.

The rate of hospital deaths was high in our cohort. In the literature the rates of hospital deaths in cancer patients vary considerably between countries, ranging from 26 to 87% [30]. In Sweden, the proportion of upper gastrointestinal cancer patients who died in hospital was 51% [31], compared to 76% in our cohort, despite the similarities between the healthcare systems in Sweden and Finland. In our cohort patients with early SPC were less likely to die in hospitals (63%) which was more in line with the Swedish figures. In addition, the likelihood of dying in SPC setting was higher in patients with early palliative care, although it remained quite low, with 19% dying in an SPC ward. Rates of patients receiving SPC who die in hospice vary in other countries, ranging from 9 to 46% [32–34]. Patients with early SPC were, however, slightly older than patients with no or late SPC, which may have had an effect on the place of death, as older patients are less likely to die in a hospital [35].

The strength of our study is the nationwide real-life data as the data was collected from the national healthcare registers, ensuring robust, nationwide perspective. The data included information from both primary and secondary healthcare, offering reliable data on healthcare resource utilization, as well as data from utilization of SPC and places of death. The limitations of the study include its retrospective nature and lack of quality-of-life data. Another limitation is the age of data, which is from 2019, as there may have been gradual changes in practices.

## Conclusion

Despite the poor prognosis and heavy symptom burden of patients with esophageal and gastric cancer, only a small proportion of patients gained access to SPC. Early initiation of SPC was associated with reduced acute healthcare utilization and hospital deaths. Hospital-at-home service was associated with increased the likelihood of home death. Although the benefits of early SPC were observed in patients with SPC contact no later than 30 days before death, the median time for the first SPC contact was earlier, 4 months before death, supporting earlier initiation of SPC as well as integration of SPC into oncological care.

## Supplementary Information

Below is the link to the electronic supplementary material.

ESM 1 (DOCX 83.8 KB)

## Data Availability

No datasets were generated or analysed during the current study.
