# Supplementary material for: Specialist palliative care is associated with reduced healthcare utilization in patients with advanced esophageal and gastric cancer: a nationwide register-based study
Source: Support Care Cancer. 2025 Jun 5;33(7):540. doi: 10.1007/s00520-025-09587-3 (PMC12141135; doi:10.1007/s00520-025-09587-3)
Supplement: Supplementary file 1 — (DOCX 83.8 KB) [file 520_2025_9587_MOESM1_ESM.docx]

**Supplement**


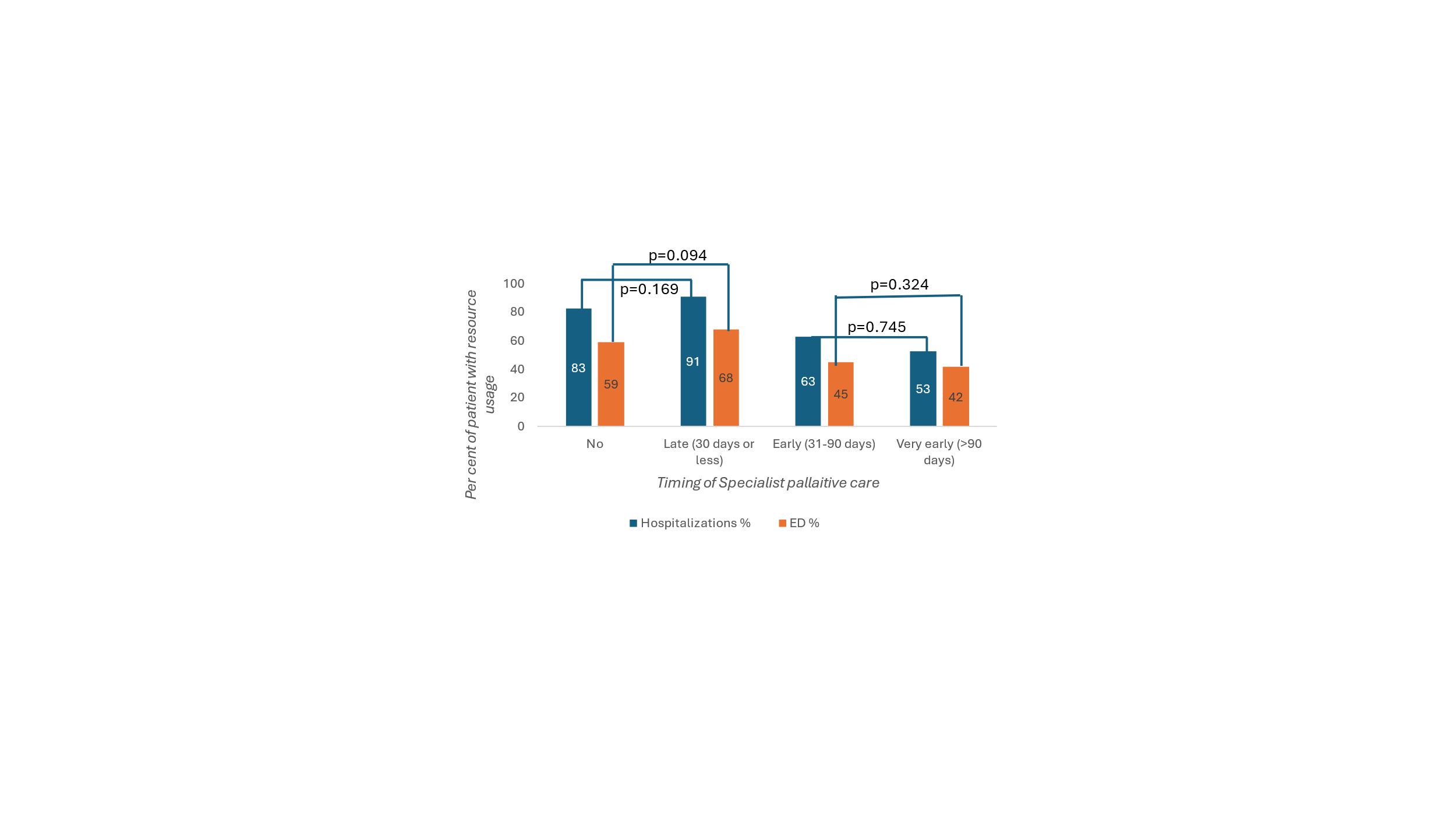


*Figure 1: Preliminary comparisons of hospitalizations and Emergency department contacts based on timing of first specialist palliative care (SPC) contact*

*Figure 2: Percentages of patients with secondary healthcare hospitalizations and Emergency department contacts over time before death. Groups are categorized by the timing of first specialist palliative care (SPC) contact (early vs. no/late SPC).*
